# Fidelity of Implementation of Oral Pre‐Exposure Prophylaxis for HIV in Public Family Planning Clinics in Kenya: Results From a Stepped‐Wedge Cluster Randomized Trial

**DOI:** 10.1002/jia2.70189

**Published:** 2026-10-08

**Authors:** Irene N. Mukui, Sandra Urusaro, John Kinuthia, Daniel Matemo, Jennifer F. Morton, Kenneth K. Mugwanya, for the FP Plus Project

**Affiliations:** ^1^ Research & Programs Kenyatta National Hospital Nairobi Kenya; ^2^ Department of Global Health University of Washington Seattle Washington USA; ^3^ Department of Clinical Operations Hospital of the University of Pennsylvania Philadelphia Pennsylvania USA; ^4^ Department of Epidemiology University of Washington Seattle Washington USA

**Keywords:** family planning clinics, implementation fidelity, integrated services, Kenya, PrEP delivery, standardized client actors

## Abstract

**Introduction:**

Integrating HIV pre‐exposure prophylaxis (PrEP) into family planning (FP) services offers a strategic opportunity to reach women at risk of HIV acquisition. We assessed fidelity to core components of oral PrEP delivery in real‐world FP clinics in Kenya.

**Methods:**

Data are from the FP Plus Project, a stepped‐wedge cluster‐randomized trial integrating oral PrEP into 12 public FP clinics in Kisumu, Kenya, where trained FP healthcare providers delivered PrEP services within routine services. Standardized unannounced client actor visits conducted between December 2021 and April 2022, and cross‐sectional exit surveys conducted between July 2021 and July 2024 among FP clients were used to evaluate whether the core components of PrEP services were delivered as intended and to assess service quality. Client actors were trained on three case scripts depicting young women in different scenarios: PrEP initiation, PrEP continuation and PrEP during routine FP visits. After each unannounced visit, client actors completed checklists documenting delivery of PrEP services, including behavioural risk assessment, HIV testing, HIV prevention and PrEP counselling, safety assessment, PrEP dispensing and quality‐of‐care. Data were analysed descriptively.

**Results:**

Overall, 28 client actor visits were completed across participating FP clinics; 12 initiation, 12 continuation and 4 integrated FP–PrEP initiation. Providers assessed at least one HIV risk behaviour in 85.7% (24/28) of visits and discussed at least one HIV prevention strategy in all visits (100%, 28/28). At least one PrEP counselling topic was discussed in 64.3% (18/28) of visits, and one medical safety area was assessed in 42.9% (12/28) of visits. Unprompted PrEP promotion was low at 28.6% (8/28) of visits. Completion of individual items within service components varied widely. Providers were respectful, non‐judgemental and used easy‐to‐understand language in all (28/28) client actor encounters. Of 1801 exit interview participants, 99.8% (1797/1801) discussed at least one HIV prevention strategy and 96.5% (1737/1801) expressed trust in the FP providers.

**Conclusions:**

Integration of oral PrEP provision into routine FP clinics was achieved with varied fidelity across service components. While engagement occurred across PrEP service components, gaps remained in completeness of counselling, PrEP promotion and medical safety assessment, highlighting the need for continued provider support and quality improvement.

**Clinical Trial Number:**

NCT04666792

## 1 Introduction

Since the World Health Organization (WHO) recommended oral pre‐exposure prophylaxis (PrEP) for persons at substantial risk of HIV in 2015, implementation has expanded to more than 150 countries, many in sub‐Saharan Africa (SSA), where the burden of HIV is highest [1, 2]. To increase access, countries have utilized different service‐delivery models and settings, initially integrating PrEP delivery within HIV care and treatment clinics [3, 4], and later expanding to sexual and reproductive health (SRH) services [5, 6], adolescent and youth health services [7], and services for key populations [8].

For women and girls in sub‐Saharan Africa who bear the greatest burden of new HIV acquisitions, expanding access to PrEP remains a priority [9]. Family planning (FP) clinics, widely accessed by women of reproductive age, provide an efficient and strategic platform for delivery of HIV PrEP, and can reduce missed opportunities for HIV prevention for women at risk [10]. In Kenya, FP services are delivered through public, private for‐profit, faith‐based and not‐for‐profit non‐governmental health facilities, with 85% of public health facilities offering FP services [11]. In 2022, 6.4 million women accessed modern contraception, 75% from the public sector [12]. FP services are primarily delivered by nurses and clinical officers, and task‐shifting is widely practised [13].

Integrating new services within existing health‐system structures is essential for sustainability and has gained more attention with declining external health funding [14]. Kenyan health policies support two‐way integration of HIV and SRH services; for example, HIV testing is routinely provided in maternal and reproductive health clinics [15, 16]. However, wider implementation is inconsistent due to health system challenges including limited provider training, staffing shortages, and fragmented commodity supply chains and data systems [17]. Effective integration requires not only policy, but system support to ensure that interventions are delivered with sufficient quality and consistency to achieve their intended outcome [18].

Fidelity, the degree to which an intervention is delivered as originally intended, is an important implementation science outcome, encompassing five dimensions: adherence to intervention, exposure or dosage, quality of delivery, participant responsiveness and programme differentiation [18]. High fidelity helps maintain intervention effectiveness during implementation, and its measurement could help distinguish between intervention and implementation failure [19, 20].

Within a large stepped‐wedge cluster randomized trial integrating oral PrEP delivery into FP clinics in Kenya, we used unannounced standardized client (SC) actors and client exit surveys to assess delivery of key components of PrEP services and the quality of care. Standardized client actors have been shown to provide objective, valid and reliable measures of provider practice and quality of care in diverse clinical settings, while reducing observer and social‐desirability bias [21, 22, 23].

## 2 Methods

### 2.1 Study Design and Setting

Data are from the Family Planning Plus (FP Plus) Project, a pragmatic stepped‐wedge cluster‐randomized trial designed to integrate provision of oral PrEP into FP services in Kisumu County, Kenya [24]. Between March 2021 and December 2023, 12 public FP clinics sequentially transitioned from usual care to offer integrated PrEP intervention in three randomized steps, each 3 months apart. Clinics were selected purposively in consultation with the County Department of Health based on volume of women accessing care, willingness to participate and geographical representation. All 12 clinics participated in the fidelity assessment, including three urban, five peri‐urban and four rural facilities across multiple care levels: health centre (1), subcounty hospitals (9), county referral hospital (1) and national referral hospital (1) [24].

### 2.2 Overview of Implementation Strategies

Detailed methods for the overall project are published elsewhere [24]. Integration of PrEP into FP clinics was supported through FP provider training and technical support. Prior to clinics transitioning to the intervention, facility‐based training on PrEP delivery was conducted for 2 hours every day over 2 weeks, using the Kenyan Ministry of Health PrEP interactive case‐based curriculum. Content was tailored to FP clinic settings and included refreshers on FP methods. Refresher sessions occurred every 3 months due to frequent staff turnover [24]. A total of 195 healthcare providers were trained (6−25 per site) across the 12 FP clinics; 57.4% (112/195), 21.5% (42/195), 7.2% (14/195) and 6.2% (12/195) of whom were nurses, HIV testing counsellors, pharmacy technicians and clinical officers, respectively.

Technical support consisted of ongoing mentoring and coaching of healthcare workers provided by study nurses who had experience in PrEP provision. The support team visited clinics weekly in the first 3 months of the intervention, and monthly thereafter. Visits entailed reviewing best practices and clinic‐level innovations to optimize implementation. Both training and technical support were withdrawn after each clinic had reached 12 months of implementation [24].

All components of PrEP services were delivered by FP clinic staff. Oral PrEP is standard of care and was made available free to clients.

### 2.3 Fidelity Study Procedures

Fidelity assessment was conducted after study clinics transitioned to the intervention period. Pre‐intervention baseline fidelity assessment was not conducted as PrEP services were not routinely offered before the study. We used two complementary approaches to evaluate fidelity of delivering core components of PrEP service as per the Kenyan national guidelines [25, 26]. These included unannounced SC actor visits conducted between December 2021 and April 2022, and client exit surveys conducted between July 2021 and July 2024, among women visiting the 12 participating FP clinics. Two fidelity dimensions were evaluated: adherence to intervention, and quality of delivery. Because the Kenyan guidelines do not distinguish which service components are more essential, fidelity was assessed equally without weighting.

#### 2.3.1 Standardized Client Actors

Standardized client actors were adolescent girls and young women (AGYW) selected and trained to portray young women at risk for HIV. Actors were trained on three case scripts representing different client scenarios: PrEP initiation, PrEP refill (continuation) and HIV risk screening during an FP visit. To ensure consistency in case presentation, actors were assigned to specific case scripts; one actor for PrEP initiation, one for PrEP refill and one for integrated FP–PrEP initiation visits. Facilities were visited by one SC actor per case type at a time, and clinics were unaware of any SC actors’ visits. Client actors were instructed to maintain a consistent demeanour, respond as per assigned case, avoid volunteering unsolicited information and initiate HIV prevention discussions when contextually appropriate following cues in the script if the provider did not initiate discussion (e.g. after receiving FP ask about “a drug that protects against HIV,” or “Is there anything I can do to help protect myself from HIV?”).

#### 2.3.2 Case Scripts

We used three standardized case scripts representing common client scenarios in PrEP service delivery.

Case 1: **PrEP initiation visit**

A 19‐year‐old woman seeking oral contraceptives, with difficulty negotiating condom use with a boyfriend of unknown HIV status. This case assessed provider performance initiating PrEP for an individual with ongoing HIV acquisition risk.

Case 2: **PrEP continuation/refill visit**

A 21‐year‐old woman returning for refill of both PrEP and oral contraceptive pills, who had difficulty negotiating condom use with a boyfriend of known HIV‐positive status. This case assessed fidelity of continuing PrEP services for an individual with persistent risk.

Case 3: **FP** **and PrEP initiation visit**

A 30‐year‐old woman seeking oral contraceptives who reported multiple partners, inconsistent condom use and uncertainty about her partners’ HIV status. This case assessed whether providers proactively screened for HIV risk and offered HIV prevention counselling, including PrEP initiation or referral, during routine FP visits.

#### 2.3.3 Client Actor Checklist

Following each encounter, client actors completed a structured checklist (Appendix S1) comprising a technical checklist and a quality of services checklist. The technical checklist was based on the Kenyan Ministry of Health PrEP guidelines' package of services which includes behavioural risk assessment, HIV testing, PrEP promotion and counselling, HIV prevention counselling, medical safety assessment, dispensing, and follow‐up [27]. Client actors indicated whether services were discussed or provided during the visit. The quality‐of‐service delivery checklist captured provider and client interaction using 4‐point Likert‐scale items (ranging from *strongly disagree* to *strongly agree*) and assessed provider communication, client privacy and respect, encouragement to ask questions, if sufficient HIV prevention information and support for additional needs were offered (Appendix S1).

#### 2.3.4 Client Exit Interviews

As part of the overall project, cross‐sectional exit surveys were administered to consecutive women exiting FP clinics on randomly selected days during the intervention phase, to characterize client experiences. Within the exit survey, items to evaluate fidelity and quality of service were added, including themes on the primary reason for visiting the FP clinic, the type of HIV prevention services discussed or provided, trust in providers and overall satisfaction with services received. Study clinics were unaware of the schedule of client exit interviews.

### 2.4 Data Analysis

We used descriptive statistics to summarize the proportion of SC visits in which each PrEP delivery component was completed or offered by the service provider. The analysis assessed completion of individual items within each service component, and whether at least one item within each service component was completed, as an indicator of provider engagement in that component. Communication and service quality scores were summarized using medians and interquartile range (IQR), and Likert scale (1–4) distribution as proportions. Negatively phrased items were reverse‐coded. Sociodemographic characteristics of exit survey participants and proportions of clients reporting receipt of HIV prevention and PrEP counselling services at FP visits, and trust in FP providers were descriptively summarized.

All analyses were conducted using R (version 4.2.1 Copyright (C) 2022; R Foundation for Statistical Computing, Vienna, Austria).

### 2.5 Ethical Approval

The FP Plus project was approved by the Kenyatta National Hospital–University of Nairobi Ethics Research Committee (P446/08/2020) and the University of Washington Human Subjects Division (STUDY00009583). Because the intervention was implemented within routine FP services and considered minimal risk, abstraction of routine programme data and client exit interviews without written consent was approved, and oral consenting used for client exit surveys (Appendix S2; IRB approval). Standardized client actor activities were included in the approved protocol, and participating actors provided written informed consent for their roles.

## 3 Results

### 3.1 Study Population and Overall Project

Between March 2021 and December 2022, 25,457 women without HIV made 28,729 FP clinic visits across the 12 participating clinics. The primary results of the main project are reported elsewhere [24]. The median age was 27 years (IQR 23–31), and >98% were using a modern contraceptive method at their first encounter. After introduction of the intervention, the proportion of visits at which women were screened for HIV risk and PrEP eligibility increased from 2.9% (213/7386) during the pre‐intervention period to 91.6% (19,560/21,343) during the intervention period. Among those eligible, oral PrEP initiation rose from 4.2% (5/120) in the pre‐intervention period to 40.6% (985/2429) during the intervention phase.

### 3.2 Fidelity Assessment Results

#### 3.2.1 Standardized Client Actor Visits

A total of 28 SC actor visits were conducted across the 12 FP clinics, including 12 PrEP initiation visits (case script 1), 12 PrEP refill (continuation) visits (case script 2) and 4 integrated FP−PrEP initiation visits (case script 3). The clinics received one visit per case type. All visits for a given case script were conducted by the same actor, except for one study clinic due to logistical issues (Table 1).

**TABLE 1:** Assignment of standardized client actors by visit type during unannounced visits in family planning clinics in Kisumu County, Kenya (*N* = 28).

Case script—visit type	Number and type of visits per SC actor	Total visits
Case 1—PrEP initiation	Actor 1 (11); Actor 2 (1)	12
Case 2—PrEP refill (continuation)	Actor 3 (11); Actor 4 (1)	12
Case 3—FP–PrEP integrated initiation	Actor 5 (3); Actor 6 (1)	4
Total		**28**

*Note*: This table shows the distribution of standardized client actors across case scripts and visit types during 28 unannounced visits to 12 public family planning clinics in Kisumu County, Kenya. Actors were assigned specific case scripts, with one actor assigned to each case type to ensure consistency in case presentation. Facility 2 was an exception, where different actors (Actors 2, 4 and 6) implemented different case scripts. Only one actor visited a facility at a time.

Abbreviations: FP, family planning; PrEP, pre‐exposure prophylaxis; SC, standardized client.

#### 3.2.2 Behavioural Risk Assessment and HIV Prevention Counselling

Based on whether providers engaged in risk assessment (defined as assessing at least one risk factor), providers assessed at least one risk factor in 85.7% (24/28) of client actor visits; less frequently in PrEP refill visits (66.7%) compared to initiation visits (100%) (Table 2). Assessment of individual risk factors varied; providers most frequently assessed partner HIV status ([75.0%], 21/28), inconsistent condom use ([42.9%], 12/28) and recent sexually transmitted infection (STI) diagnosis ([42.9%], 12/28) (Table 2). Similar patterns of risk factors assessed were observed across visit types, with lower assessment during PrEP refill visits.

**TABLE 2:** Fidelity of oral pre‐exposure prophylaxis service delivery components across standardized client actor simulated initiation, refill, and integrated PrEP and family planning visits in 12 public FP clinics in Kisumu County, Kenya (*N* = 28 standardized client visits).

PrEP service delivery component	Individual factors/areas	PrEP initiation (*n* = 12)	PrEP refill/continuation (*n* = 12)	FP + PrEP initiation (*n* = 4)	All visits (*n* = 28)
**Behavioural risk assessment**	**≥1 specific risk assessed**	12 (100.0)	8 (66.7)	4 (100.0)	24 (85.7)
	Sex partner(s) with high risk and HIV status is unknown	10 (83.3)	7 (58.3)	4 (100.0)	21 (75.0)
	Condom use assessed	6 (50.0)	2 (16.7)	4 (100.0)	12 (42.9)
	Recent STI diagnosis	7 (58.3)	4 (33.3)	1 (25.0)	12 (42.9)
	Recurrent PEP use	1 (8.3)	1 (8.3)	2 (50.0)	4 (14.3)
	History of intimate partner violence	2 (16.7)	0 (0.0)	1 (25.0)	3 (10.7)
	Transactional sex assessed	1 (8.3)	0 (0.0)	0 (0.0)	1 (3.6)
	Injection drug use	1 (8.3)	0 (0.0)	0 (0.0)	1 (3.6)
	HIV serodiscordant partnership	0 (0.0)	0 (0.0)	2 (50.0)	2 (7.1)
	Recurrent sex under alcohol/drugs	0 (0.0)	1 (8.3)	0 (0.0)	1 (3.6)
**HIV prevention counselling**	**≥1 prevention strategy discussed**	12 (100.0)	12 (100.0)	4 (100.0)	28 (100.0)
	Know your partner's status	12 (100.0)	7 (58.3)	3 (75.0)	22 (78.6)
	Condom counselling	7 (58.3)	2 (16.7)	4 (100.0)	13 (46.4)
	Partner testing offered	0 (0.0)	0 (0.0)	0 (0.0)	0 (0.0)
	Asked about STI symptoms	5 (41.7)	3 (25.0)	1 (25.0)	9 (32.1)
	Discussed circumcision for male partners	0 (0.0)	0 (0.0)	0 (0.0)	0 (0.0)
	Provided information about PEP	2 (16.7)	0 (0.0)	2 (50.0)	4 (14.3)
**PrEP promotion**	Provider asked about interest in PrEP without prompting	4 (33.3)	0 (0.0)	4 (100.0)	8 (28.6)
**PrEP counselling**	**≥1 PrEP counselling topic**	10 (83.3)	4 (33.3)	4 (100.0)	18 (64.3)
	PrEP works when taken daily	9 (75.0)	3 (25.0)	4 (100.0)	16 (57.1)
	PrEP is safe	4 (33.3)	1 (8.3)	1 (25.0)	6 (21.4)
	Side effects and how to reduce	1 (8.3)	0 (0.0)	1 (25.0)	2 (7.1)
	Need to repeat HIV testing when on PrEP	2 (16.7)	3 (25.0)	2 (50.0)	7 (25.0)
	PrEP does not protect against STI/pregnancy	3 (25.0)	0 (0.0)	2 (50.0)	5 (17.9)
	PrEP used during risk periods only	3 (25.0)	1 (8.3)	3 (75.0)	7 (25.0)
**HIV testing offered**		12 (100.0)	12 (100.0)	4 (100.0)	28 (100.0)
**PrEP dispensing**					
	Received PrEP medication during visit	9 (75.0)	11 (91.7)	3 (75.0)	23 (82.1)
	PrEP dispensed in FP clinic room	7 (58.3)	9 (75.0)	0 (0.0)	16 (57.1)
	Provided PrEP refill date	9 (75.0)	10 (83.3)	2 (50.0)	21 (75.0)
**Medical safety assessment**	**≥1 assessed**	6 (50.0)	4 (33.3)	2 (50.0)	12 (42.9)
	Screened for HIV symptoms and signs	0 (0.0)	0 (0.0)	0 (0.0)	0 (0.0)
	Asked about comorbidities	2 (16.7)	0 (0.0)	0 (0.0)	2 (7.1)
	Asked about other medical conditions	1 (8.3)	0 (0.0)	1 (25.0)	2 (7.1)
	Asked if client pregnant or breastfeeding	5 (41.7)	4 (33.3)	1 (25.0)	10 (35.7)

*Note*: This table presents the proportion of standardized client visits during which FP providers delivered specific components of PrEP service. Data are based on 28 unannounced standardized client visits conducted in 12 public FP clinics in Kisumu County, Kenya, between December 2021 and April 2022. Data are presented for each visit type and then overall for all visits combined.

Abbreviations: FP, family planning; HIV, human immunodeficiency virus; PEP, post‐exposure prophylaxis; PrEP, pre‐exposure prophylaxis; STI, sexually transmitted infection.

For HIV prevention counselling, all client actor visits ([100%], 28/28) included discussion on at least one HIV prevention strategy (Table 2). The most common strategies discussed included the need to know partner's HIV status, condom use, and discussion on STIs in 78.6% (22/28), 46.4% (13/28) and 32.1% (9/28) of visits, respectively. No visits included discussion on benefits of male partner circumcision or provision of partner HIV testing resources (Table 2).

#### 3.2.3 PrEP Promotion, PrEP Counselling, HIV Testing

For PrEP promotion, health providers proactively asked client actors if they were interested in PrEP without prompting in 28.6% (8/28) of the visits (Table 2); occurring in all integrated FP‐PrEP visits ([100.0%], 4/4), and in 33.3% of initiation visits (4/12).

Counselling on at least one PrEP‐related topic occurred in 64.3% (18/28) of visits; more frequently during PrEP initiation (83.3%) and integrated FP/PrEP (100%) visits than in refill visits (33.3%) (Table 2). The most frequently discussed PrEP topic was PrEP works when taken daily ([57.1%], 16/28). HIV testing was offered during all client actor visits ([100%], 28/28).

#### 3.2.4 PrEP Dispensing

Oral PrEP dispensing occurred in 82.1% (23/28) of visits (Table 2) and was higher during refill visits (91.7%) than in the initiation visits (75.0%). PrEP was dispensed in the FP clinic room in 57.1% (16/28) of encounters, and a refill return date was given in 75.0% (21/28) of visits (Table 2).

#### 3.2.5 Medical Safety Assessment

Providers discussed at least one medical safety issue in 42.9% (12/28) of visits (Table 2). The most common safety assessment was asking about pregnancy or breastfeeding; 35.7% (10/28) (Table 2), while assessment of comorbidities and other medical conditions was infrequent. No encounters included screening for acute HIV acquisition (0/28) (Table 2).

#### 3.2.6 Fidelity of PrEP Service Delivery by Visit Type

When compared across visit types, PrEP refill visits had lower coverage of behavioural risk assessment, and HIV prevention and PrEP counselling compared to initiation visits, while PrEP dispensing was higher in the refill visits. Although coverage varied by visit type, the specific risk factors assessed and counselling topics most frequently discussed were similar across visit types (Table 2).

#### 3.2.7 Communication and Quality of Service Delivery

Client actors reported that providers used easy‐to‐understand language and were non‐judgemental in all encounters (100% agree) (Figure 1). Providers were respectful in all encounters (71% strongly agree and 29% agree) and listened without interruption in most encounters (89% strongly agree, 7% agree) (Figure 1). Few client actors (11% strongly agree, 14% agree) felt encouraged to ask questions or received information (14% strongly agree) on where to seek additional support. The median Likert scores (IQR) are summarized in Table S1.

**FIGURE 1:**
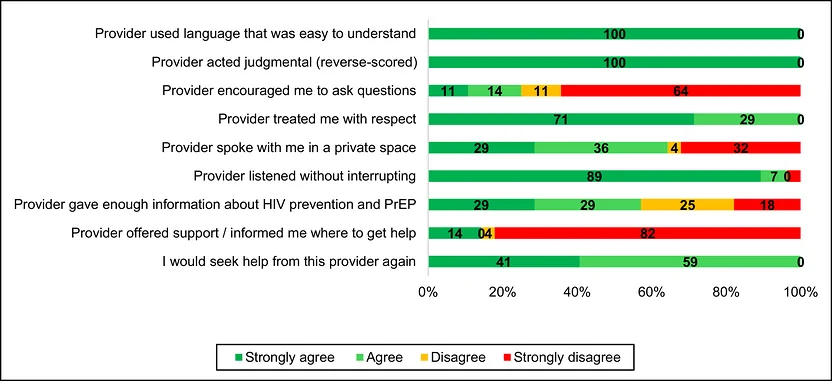
Client‐reported quality of oral pre‐exposure prophylaxis service delivery during standardized client visits in 12 family planning clinics in Kisumu County, Kenya (*N* = 28 visits). This figure presents standardized client assessments of provider communication and quality of care during 28 unannounced visits to 12 public family planning clinics in Kisumu County, Kenya. Measures are based on Likert‐scale responses (1 = strongly disagree, 2 = disagree, 3 = agree, 4 = strongly agree) evaluating provider behaviour, including clarity of communication, respect, privacy and encouragement of client engagement. Bars represent the proportion of visits corresponding to each response category for each quality‐of‐care indicator. HIV, human immunodeficiency virus; PrEP, pre‐exposure prophylaxis; SC, standardized client.

### 3.3 Client Exit Interviews

#### 3.3.1 Participant Characteristics

A total of 1801 women receiving FP services participated in the cross‐sectional client exit interviews. The median age was 27 years (IQR, 23–32), 36.9% (665/1801) were aged 15–24 years and 61.5% (1108/1801) were married. Nearly all women (97.5%; 1756/1801) reported the primary reason for visiting the clinic as seeking contraception services, while 14.7% (264/1801) reported seeking HIV prevention services.

#### 3.3.2 HIV Prevention and PrEP Counselling

Nearly all clients (99.8% [1797/1801]) reported discussing at least one HIV prevention service with the FP provider (Table 3). The most frequently discussed topics were counselling on PrEP use (75.1% [1353/1801]), HIV testing (64.2% [1156/1801]) and HIV risk‐reduction counselling (57.9% [1043/1801]) (Table 3).

**TABLE 3:** Client‐reported receipt of HIV prevention and pre‐exposure prophylaxis counselling services during family planning clinic visits among 1801 women in Kisumu County, Kenya.

Service delivery component	Clients reporting topic was discussed Total = 1801 *n*	Proportion %
**HIV prevention and PrEP counselling discussed**		
Discussed at least one HIV prevention service	**1797**	**99.8**
**Specific HIV prevention topics discussed** ^a^		
HIV testing	1156	64.2
HIV risk‐reduction counselling	1043	57.9
STI risk counselling	622	34.5
Testing and treatment of STIs	230	12.8
Assessed and counselled on oral PrEP	1353	75.1
Partner HIV testing or status	358	19.9
VT counselling	37	2.1
PEP counselling	34	1.9
Condom‐use counselling	333	18.5

*Note*: This table summarizes client‐reported indicators of HIV prevention and pre‐exposure prophylaxis service delivery based on cross‐sectional exit interviews conducted among women attending 12 public family planning clinics in Kisumu County, Kenya, between July 2021 and July 2024. The data reflect what HIV prevention and PrEP counselling topics were discussed by the provider during family planning visits. Percentages are based on the number of respondents for each indicator (*N* = 1801).

Abbreviations: HIV, human immunodeficiency virus; PEP, post‐exposure prophylaxis; PrEP, pre‐exposure prophylaxis; STI, sexually transmitted infection; VT, vertical transmission.

^a^ Data are not mutually exclusive; participants may report more than one service or topic discussed.

#### 3.3.3 Trust in Providers and Satisfaction With Clinic Experience

Overall, 96.5% reported trust in FP providers to provide correct HIV prevention information (70.6% [1271/1801] full trust and 25.9% [466/1801] some trust). Most women (93.6%, 1686/1801) reported being satisfied with their FP clinic experience.

## 4 Discussion

This study assessed fidelity to delivery of oral PrEP services integrated into routine FP clinics and delivered by public healthcare workers. Fidelity was assessed for individual items within each component, and provider engagement with the service component based on completion of at least one item. Engagement with core PrEP service components was high (>80%) for behavioural risk screening, HIV prevention counselling and PrEP dispensing, but low for PrEP counselling, PrEP promotion and medical safety assessment. We found great variability in assessment of individual risk factors, counselling on specific topics in HIV prevention and on PrEP, and medical safety assessments, with about half of the individual items completed in fewer than half of the encounters. Fidelity also varied by visit type; PrEP refill visits had less behavioural risk assessment and HIV prevention counselling compared to PrEP initiation visits, while PrEP dispensing was higher in refill visits, a finding that reflects common clinical practice, where refill visits tend to focus on medication continuation and adherence. Findings from the client exit interviews on HIV prevention and PrEP counselling were consistent with client actor assessments. Nearly all women reported discussion of at least one HIV prevention service (99.8%); however, as with client actor findings, there was variability in coverage of specific counselling topics.

We found important fidelity gaps in assessment of individual behavioural risks, in HIV prevention and PrEP counselling and in unprompted promotion of PrEP by providers. Providers infrequently addressed key aspects of HIV prevention, including condom use, STI presence and partner services. Counselling on PrEP side effects and what PrEP cannot prevent was also limited. While PrEP dispensing occurred in most encounters, failure to dispense PrEP in all eligible scenarios was a missed prevention opportunity. Medical safety assessment, aside from pregnancy, was limited; in particular, low screening for acute HIV acquisition is a concern that needs focused provider training due to risk for PrEP use during undiagnosed HIV acquisition [27, 28]. These gaps represent missed opportunities for comprehensive HIV and STI prevention that limit realization of full benefits and reflect broader challenges; for instance, STI services are inconsistently implemented in routine settings despite policy recommendations [29, 30].

Regarding the quality of services and trust in providers, client actor assessments indicated positive provider and client interactions. Providers were respectful and non‐judgemental, and communication was clear in all or most encounters. These findings were consistent with results from exit surveys among women, who reported high satisfaction with services and trust in FP providers. Some gaps were observed, including limited encouragement to ask questions and discussions on where to seek additional support.

Previous studies have shown the feasibility of integrating PrEP into FP clinics and other reproductive health service settings, but have been primarily implemented using dedicated study staff [31]. Our study focused on fidelity of PrEP delivery by public healthcare providers, an area that has not been widely evaluated. A pharmacy‐based PrEP delivery study in Kenya using client actors reported high fidelity exceeding 80% in most items in risk assessment, HIV and PrEP counselling, and PrEP dispensing [32]. Similar to our findings, the study also found gaps in condom use counselling, partner HIV testing and medical safety assessment, including low screening for acute HIV acquisition [32]. An earlier study using mystery clients, assessing fidelity to PrEP counselling for AGYW in maternal and child health clinics in western Kenya, reported overall low adherence to counselling and risk assessment guidelines, with an adherence score of 31% [33]. Gaps were identified in counselling topics including condom use, partner HIV testing, STI assessment and PrEP side effect counselling [33]. Broader PrEP implementation studies from South Africa, Uganda and other SSA countries have highlighted challenges in maintaining consistent counselling quality and comprehensiveness of prevention services in routine settings [30, 34, 35]. Together, these findings suggest that integrating and maintaining effective integration of PrEP into routine services would require ongoing monitoring and continuous quality improvement.

In the FP Plus project, PrEP integration was supported through provider facility‐based training and ongoing technical assistance to clinics. Important gaps were identified in the fidelity of service delivery. Public health facilities in Kenya and many settings in Africa are overburdened with high client volume, limited space and staff shortages, and frequent staff transfers; and adding more services further constrains already limited clinic resources [36]. These limitations may partially explain variability in completeness of services and how providers may prioritize some service elements to be more critical than others, including prevention discussions. Although our study was conducted in public FP clinics in Kisumu County, which could limit geographic and clinical settings generalizability, the findings have broader relevance for PrEP scale‐up in resource constrained settings where integration into existing platforms is planned [17, 37].

This study had limitations. The small number of client actor visits limited further analysis, including comparisons of fidelity across visit types and facilities. In addition, because visits for each case type were conducted by a single client actor, responses are likely correlated within actors, and inter‐actor reliability could not be assessed. This also limited certainty of whether observed patterns fully reflect provider practice or actor recall or recording or both, although findings were consistent across client actor assessments and exit interviews. We did not conduct repeated fidelity assessments during implementation and, therefore, could not determine if providers’ performance improved with continued support. Lastly, although client actors received training and similar checklists have been used previously [32, 33], it is possible the length of the checklist could have contributed to recall errors.

Despite these limitations, our findings are strengthened by the fact that the fidelity assessment was embedded within a large real‐world implementation project, and the use of client actor assessments and client exit interviews was consistent and offered complementary views of fidelity of service delivery. The main trial demonstrated increased access to PrEP during the intervention, suggesting that provider capacity building and clinic support can lead to increased access to oral PrEP [24].

## 5 Conclusions

In conclusion, our study demonstrated that PrEP services can be integrated into routine FP services and delivered by public sector workers, though fidelity varied across different components of PrEP services. Providers engaged with all components of PrEP service delivery, but gaps remained in completeness and comprehensiveness of risk assessment, counselling, PrEP promotion, medical safety screening and PrEP dispensing. Women reported positive client experiences and high trust in providers. Continued training and mentoring of providers, plus periodic fidelity assessments and targeted quality improvement addressing identified service gaps are needed to strengthen the consistency and comprehensiveness of integrated PrEP services and ensure effectiveness of PrEP.

Future fidelity assessments should incorporate repeated measurements to assess changes over time, and where client actors are used, multiple visits, with multiple actors within visit types to allow comparability and improve certainty of findings. Where PrEP services are already established, baseline fidelity assessments should be included to better attribute changes following implementation strategies. Future research should also explore weighting service components according to potential consequences of non‐adherence, to strengthen fidelity interpretation.

## Author Contributions

KKM conceptualized and designed the parent study and obtained funding. KKM and JK contributed to the conception and design of this study; JFM and DM contributed to programme administration and coordination; DM contributed to data collection and supervision; INM, SU and KKM developed the analysis plan for this manuscript; INM led the statistical analysis and prepared the first draft of the manuscript. All authors contributed to project development, writing and critical revision of the paper. All authors read and approved the final manuscript.

## Funding

The FP Plus Project is supported by the National Institute of Mental Health of the US National Institutes of Health (grant nos. R01 MH123267 and R00 MH118134). The funder had no role in design, data collection and analysis, decision to publish or preparation of the manuscript.

## Conflicts of Interest

KKM has received research support from Gilead paid to his institution. All other authors declare no conflicts of interest.

## Supporting information




**Appendix S1**: FP PLUS standardized actor checklist. This is the checklist completed by client actors immediately after each provider interaction to document whether key PrEP service‐delivery actions were performed and to assess the quality‐of‐service delivery.

**Appendix S2**: University of Washington IRB APPROVAL OF APPLICATION. This IRB approval contains documentation of the waiver of written consent for client exit interviews, as requested in response to reviewer comments.

**Table S1**: Communication and quality of PrEP service delivery reported by standardized clients. The table presents the proportion of standardized‐client visits in which client actors agreed or strongly agreed (scores ≥3) with statements assessing provider performance, plus the corresponding median Likert scores and interquartile ranges (IQR).

## Data Availability

The full original dataset underpinning this manuscript can be accessed by academic researchers by contacting the International Clinical Research Center at the University of Washington (icrc@uw.edu). Data will be shared without investigator support, after approval of a proposal, with a signed data access agreement for research purposes.
