# Supplementary material for: Fidelity of Implementation of Oral Pre‐Exposure Prophylaxis for HIV in Public Family Planning Clinics in Kenya: Results From a Stepped‐Wedge Cluster Randomized Trial
Source: J Int AIDS Soc. 2026 Oct 8;29(Suppl 5):e70189. doi: 10.1002/jia2.70189 (PMC13650392; doi:10.1002/jia2.70189)
Supplement: Supplementary file 1 [file JIA2-29-e70189-s002.pdf]

IRB APPROVAL OF APPLICATION

March 19, 2020

Dear Kenneth Mugwanya:

On 3/19/2020, University of Washington IRB Committee J reviewed the following application:

Type of Review:	Initial Study
Title of Study:	PrEParing family planning clinics to streamline integration of HIV prevention services for young women in Kenya
Investigator:	Kenneth Mugwanya
IRB ID:	STUDY00009583
Funding:	Name: National Institutes of Health (NIH), Grant Office ID: A148068, Funding Source ID: R01 MH123267<br>Funding Title(s): “Integrating PrEP delivery in family planning clinics in Kenya”
IND, IDE, or HDE:	None

IRB Approval

Under FWA #00006878, the IRB approved your activity.

- **Depending on the nature of your study, you may need to obtain other approvals or permissions to conduct your research. For example, you might need to apply for access to data or specimens (e.g., to obtain UW student data). Or, you might need to obtain permission from facilities managers to approach possible subjects or conduct research procedures in the facilities (e.g., Seattle School District; the Harborview Emergency Department).**
- Your application qualified for expedited review (“minimal risk”; Categories 2a, 4, 5, 6, and 7).
- Under the Revised Common Rule this IRB approval is valid until study completion. In other words, there is no expiration date and you are not required to submit Continuing Review Reports to maintain your approval. However, you are still required to (1) obtain IRB approval before making any changes (modifications) to your research, and (2) provide the IRB with any Reportable New Information such as breaches of confidentiality or unanticipated problems.
- This approval applies only to the activities described in your application (including any references to specific grant sections). It does not include other activities that may be described in your grant or contract.
- Your study automatically has a Certificate of Confidentiality (CoC), because you have NIH funding. A description of the CoC protections and responsibilities has been placed in your study’s Documents section.
- If you plan to continue data collection past the expiration of your NIH funding and the CoC, contact the Human Subjects Division prior to the end of your funding. We will help you determine whether you need to apply for a CoC extension.

- **Be sure to consult HSD's COVID-19 webpage for updated information about the novel coronavirus and human subjects research.**

Determinations, waivers, and regulations

The IRB made the determinations and waivers listed in the table below. Note that any granted waivers of consent or parent permission do not override a subject's refusal to provide broad consent.

Requirement	Determination or Waiver
Consent	Waived to collect medical record information and patient experience surveys for HIV- uninfected women, subjects in time and motion observation, and to access data about partners who are offered home HIV tests.
Documentation of consent	Waived for women doing the SMS intervention, clinic readiness to implement survey, and survey to women who discontinue PrEP.

Location of documents

Use the consent forms that were approved and stamped by the IRB. They can be downloaded from the Final column under the **Documents tab** in Zipline.

In addition, HSD has uploaded the following documents to the **Documents tab** in Zipline:

- Certificate of Confidentiality Acknowledgement Letter

Thank you for your commitment to ethical and responsible research. We wish you great success!

Sincerely,

Jeff Love, IRB Administrator
206-543-2921, lovej2@uw.edu
