# Supplementary material for: Fidelity of Implementation of Oral Pre‐Exposure Prophylaxis for HIV in Public Family Planning Clinics in Kenya: Results From a Stepped‐Wedge Cluster Randomized Trial
Source: J Int AIDS Soc. 2026 Oct 8;29(Suppl 5):e70189. doi: 10.1002/jia2.70189 (PMC13650392; doi:10.1002/jia2.70189)
Supplement: Supplementary file 2 [file JIA2-29-e70189-s003.pdf]

Administrative

Checklist ID	__________________________________
Date	__________________________________
Facility name	◯ 10: JOOTRH<br>◯ 11: Kisumu County-Hospital<br>◯ 12: Lumumba Sub-County Hospital<br>◯ 13: Migosi Sub-County Hospital<br>◯ 14: Ahero County Hospital<br>◯ 15: Rabuor Sub-County Hospital<br>◯ 16: Muhoroni County Hospital<br>◯ 17: Chulaimbo County Hospital<br>◯ 18: Nyakach County Hospital<br>◯ 19: Manyuanda Sub-County<br>◯ 20: Airport North Health Centre<br>◯ 21: Kombewa County Hospital
Standardized patient ID	◯ 1A<br>◯ 1B<br>◯ 2A<br>◯ 2B<br>◯ 3A<br>◯ 3B
Visit ID	◯ Case1<br>◯ Case2<br>◯ Case3
Time presented at registration	__________________________________
Time in to visit provider	__________________________________
Time at exit of visit with provider	__________________________________

1. Behavioral risk assessment

1. Did the provider ask you about the following specific behaviors that might put you at risk of acquiring HIV?	◯ Yes<br>◯ No
a. Inconsistent or no condom use	◯ Yes<br>◯ No
b. Having a sex partner(s) with high risk and HIV status is unknown	◯ Yes<br>◯ No
c. Engaging in transactional sex	◯ Yes<br>◯ No

d. Ongoing intimate partner/gender based violence	◯ Yes<br>◯ No
e. Recent STI	◯ Yes<br>◯ No
f. Recurrent use of post-exposure prophylaxis (PEP)	◯ Yes<br>◯ No
g. Recurrent sex under the use of alcohol/recreational drugs	◯ Yes<br>◯ No
h. Injection drug use with shared injection equipment	◯ Yes<br>◯ No
i. In HIV serodiscordant partnership where the HIV-infected partner is not virally suppressed or they want to conceive	◯ Yes<br>◯ No

2. PrEP promotion

1. Did the provider ask if you were interested in PrEP?<br>(independent of you asking)	◯ Yes<br>◯ No

3. Provider counseling

1. Did the provider counsel on HIV prevention strategies?	◯ Yes<br>◯ No
a. Condom use	◯ Yes<br>◯ No
b. Knowing your partner's status	◯ Yes<br>◯ No
c. Providing resources to conduct partner HIV testing	◯ Yes<br>◯ No
d. Asked about STI symptoms like vaginal discharge, sores, or blisters	◯ Yes<br>◯ No
e. Benefits of circumcision for male partners	◯ Yes<br>◯ No
f. PEP	◯ Yes<br>◯ No
g. The risk of multiple sexual partners	◯ Yes<br>◯ No
2. What other HIV prevention strategies were discussed?	◯ None<br>◯ Other

Specify other HIV prevention strategies discussed ________________________

Did the provider counsel on the following PrEP topics?

	Yes	No
a. PrEP works when taken daily	◯	◯
b. PrEP is safe	◯	◯
c. PrEP has side effects and how to reduce them	◯	◯
d. HIV testing must be repeated at 1 month and every 3 months while taking PrEP and/or if experiencing HIV symptoms	◯	◯
e. PrEP does not protect against STIs or pregnancy	◯	◯
f. PrEP should be used during periods of HIV risk, not used life-long	◯	◯

3. Did the provider counsel on family planning methods?
◯ Yes
◯ No

4. Medical safety assessment

Did the provider:
1. Screen for signs and symptoms of HIV infection
◯ Yes
◯ No

2. Ask about comorbidities like hypertension, kidney disease or diabetes
◯ Yes
◯ No

3. Ask about any other known medical conditions
◯ Yes
◯ No

4. Ask if currently pregnant or breastfeeding
◯ Yes
◯ No

3. HIV testing

1. Did the provider test you for HIV?
◯ Yes
◯ No

a. If yes, where was HIV testing done?
◯ FP clinic
◯ Not in FP clinic

2. Did the provider conduct counselling before the HIV test?
a. HIV information
◯ Yes
◯ No

b. Risks and how to reduce them
◯ Yes
◯ No

c. How the test works	◯ Yes<br>◯ No
3. Was the testing assisted HIV self-testing?	◯ Yes<br>◯ No
4. Did you test in a private room?	◯ Yes<br>◯ No
5. Did the provider conduct counselling after providing HIV test results?	◯ Yes<br>◯ No
a. Your test results and what they mean	◯ Yes<br>◯ No
b. A plan to seek further care (if positive) or reduce HIV risk (if negative)	◯ Yes<br>◯ No

4. PrEP dispensing

1. Did you receive PrEP medications at the end of your visit?	◯ Yes<br>◯ No
a. If yes, where was dispensing done?	◯ FP clinic<br>◯ Pharmacy
2. How many months of PrEP medication did you receive?	__________________________________
3. Did the provider give you a date to come back to the clinic for PrEP refills?	◯ Yes<br>◯ No
Did you feel that the provider knew you were a standardized patient?	◯ Yes<br>◯ No
Please describe	__________________________________

COMMUNICATION ASSESSMENT

5. Quality of services

The provider:

	Strongly disagree	Disagree	Agree	Strongly agree
1. Used language that was easy to understand	◯	◯	◯	◯
2. Acted judgmental towards me	◯	◯	◯	◯

3. Encouraged me to ask questions	◯	◯	◯	◯
4. Treated me with respect	◯	◯	◯	◯
5. Spoke with me in a private space (e.g. no other people in the room, door closed)	◯	◯	◯	◯
6. Listened to me without interrupting	◯	◯	◯	◯
7. Gave me enough information about HIV prevention and PrEP	◯	◯	◯	◯
8. Offered me support or informed me on where to get support for my other needs	◯	◯	◯	◯
9. I would seek help from this provider again	◯	◯	◯	◯

Data Entry Date ________________________

Staff ID/Initials ________________________
