# Supplementary material for: Fidelity of Implementation of Oral Pre‐Exposure Prophylaxis for HIV in Public Family Planning Clinics in Kenya: Results From a Stepped‐Wedge Cluster Randomized Trial
Source: J Int AIDS Soc. 2026 Oct 8;29(Suppl 5):e70189. doi: 10.1002/jia2.70189 (PMC13650392; doi:10.1002/jia2.70189)
Supplement: Supplementary file 3 [file JIA2-29-e70189-s001.docx]

**Supporting information**

**Supplemental Table 1: Standardized client assessments of provider communication and quality of oral PrEP service delivery reported by standardized clients during visits to family planning clinics in Kisumu County, Kenya (N = 28)**

| **Indicator of quality; N= 28** | **N (Likert score ≥3)** | **%** | **Median Likert score (IQR)** |
| --- | --- | --- | --- |
| Provider used language that was easy to understand | 28 | 100.0 | 4 (4–4) |
| Provider acted judgmentally towards me (reverse scored) | 28 | 100.0 | 4 (4–4) |
| Provider encouraged me to ask questions | 7 | 25.0 | 1 (1–2.25) |
| Provider treated me with respect | 28 | 100.0 | 4 (3–4) |
| Provider spoke with me in a private space | 18 | 64.3 | 3 (1–4) |
| Provider listened to me without interrupting | 27 | 96.4 | 4 (4–4) |
| Provider gave enough information about HIV prevention and PrEP | 16 | 57.1 | 3 (2–4) |
| Provider offered support or informed me where to get help | 4 | 14.3 | 1 (1–1) |
| I would seek help from this provider again ^†^ | 27 | 100.0 | 3 (3–4) |

***Legend supplemental table 1:*** *^†^one response was missing*

*The table presents the proportion of standardized client visits in which SCs agreed or strongly agreed (scores ≥ 3) with statements assessing provider performance, plus the corresponding median Likert scores and interquartile ranges (IQR).* *Likert-scale responses ranged from 1 = strongly disagree to 4 = strongly agree. Negatively phrased items were reverse scored so that higher scores reflected more positive provider performance.* *Abbreviations: PrEP = pre-exposure prophylaxis; SC = standardized client; IQR = interquartile range.*
